# Editorial: Neonatal and pediatric brain injury: novel therapeutics and perspective

**DOI:** 10.3389/fped.2023.1210749

**Published:** 2023-05-16

**Authors:** Takeo Mukai, Rafael Galindo, Jacques-Olivier Coq

**Affiliations:** ^1^ Department of Pediatrics, The University of Tokyo Hospital, Tokyo, Japan; ^2^ Department of Neurology and Hope Center for Neurological Disorders, Washington University School of Medicine, St Louis, MO, United States; ^3^ Institut des Sciences du Mouvement, UMR7287, Aix-Marseille Université, Marseille, France; ^4^ Centre National de la Recherche Scientifique (CNRS), UMR7287, Marseille, France

**Keywords:** neonatal brain injury, pediatric brain injury, traumatic brain injury, stroke, hypoxic-ischemic encephalopathy

**Editorial on the Research Topic** Neonatal and pediatric brain injury: novel therapeutics and perspective

Neonatal and pediatric brain injuries have several causes, such as traumatic injury, stroke, hypoxic-ischemic encephalopathy (HIE), intraventricular hemorrhage (IVH), systemic inflammation and infection, and they place a great burden not only on the infant but also on the family. Several conventional therapies are used to treat brain injuries and their therapeutic effects have been established to a certain extent. However, complete neurological recovery is still difficult, and neurological sequelae often persist. Therefore, novel treatments are required to treat such injuries. Many basic research and clinical studies using new drugs, cell therapy, and rehabilitation/interventions have been conducted and their feasibility and efficacy have been reported. Recent advances in novel therapeutics are promising for the treatment of neonatal and pediatric brain injuries. Balanced and comprehensive expertise in developmental therapeutics is required to provide appropriate treatment for neonates and children. The purpose of this special issue was to conduct research on new therapeutics for neonatal and pediatric brain injuries in the seeding stage, which is key to new treatment strategies that can be developed into clinical research in the future. This issue also aims to bring together basic and clinical evidence for many of the treatments currently underway for brain injury to establish a more robust treatment protocol.

For basic research to understand the mechanism of HIE, Zen et al. elucidated that the ambient temperature is useful for creating a rodent model with the appropriate severity of targeted neuropsychological symptoms to establish a novel therapy for HIE. The pathophysiology of HIE has been previously studied in several rodent models to develop novel treatments (1). Although it is well known that a high ambient temperature results in severe HIE (2), the effect of subtle changes in ambient temperature during a hypoxic-ischemic insult has not been fully studied. Zen et al. revealed that even a small gradual change of 0.5°C produced significant differences in pathological and behavioral changes and contributed to the accumulation of microglia. This basic HIE experimental animal model is very important for future clinical applications.

IVH is a common complication in preterm infants related to neurodevelopmental outcomes, similar to HIE. Infants with severe IVH are at a higher risk of adverse neurological outcomes and death (3). Wang et al. conducted a prospective cohort study to evaluate the effect of different degrees of IVH on mortality and neurodevelopmental outcomes in preterm infants. As a result, 1,079 preterm infants were included; 380 (35.2%) had grade I–II IVH, 74 (6.9%) had grade III–IV IVH, and 625 (57.9%) did not have IVH. After adjusting for confounding factors, preterm infants with III–IV IVH had higher rates of cerebral palsy (CP) [26.7% vs. 2.4%, OR = 6.10, 95% confidence interval (CI) (1.840–20.231), *p* = 0.003], disability [43.3% vs. 13.9%, OR = 2.49, 95% CI (1.059–5.873), *p* = 0.037], death [55.2% vs. 20.1%, OR = 3.84, 95% CI (2.090–7.067), *p* < 0.001], and disability + death [73.7% vs. 28.7%, OR = 4.77, 95% CI (2.518–9.021), *p* < 0.001] compared to those without IVH. However, the mortality and incidence of neurodevelopmental disability in infants with stage I–II IVH were similar to those in infants without IVH (*p* > 0.05). They demonstrated that preterm infants of <30 weeks’ gestational age with I–II IVH had similar neurological outcomes and mortality compared to those without IVH, while III–IV IVH was associated with CP, disability, death, and disability + death at 18–24 months corrected age.

Leifsdottir et al. also focused on neurodevelopmental disability in preterm infants and investigated the cerebrospinal fluid (CSF) proteome in them. They examined whether a specific proteomic profile in the CSF of preterm infants differed from that of term infants and whether there were novel biomarkers of neurodevelopmental outcomes in preterm infants. Increased levels of brain-specific proteins associated with neurodevelopment and neuroinflammatory pathways made up a distinct protein profile in preterm infants. The most significant differences in the protein profiles were observed in proteins involved in the neurodevelopmental regulation and synaptic plasticity, as well as components of the innate immune system. Among the proteins that provided strong predictors of outcomes were vascular endothelial growth factor C, Neurocan core protein, and seizure protein 6, all of which were highly important in normal brain development. This study contributes to our understanding of neurodevelopmental processes in the preterm brain and opens up opportunities for tentative treatment options for preterm infants, including supplementary proteins or protein substrates.

Zhu et al. focused on traumatic brain injury, and investigated the predictive value of inflammation-based scores combined with radiological characteristics in children with moderate or severe traumatic brain injury (MS-TBI). A total of 104 pediatric patients with MS-TBI were retrospectively enrolled and randomly divided into training and validation cohorts in a 7:3 ratio. A prognostic nomogram was constructed and its predictive performance was validated in both the training and validation cohorts. Sex, platelet-to-lymphocyte ratio at admission, and basal cistern status from the initial CT findings were identified as independent prognostic predictors of MS-TBI in multivariate logistic analysis. Based on these findings, a nomogram was then developed, and its concordance index values were 0.918 [95% CI: 0.837–0.999] in the training cohort and 0.86 [95% CI: 0.70–1.00] in the validation cohort. They concluded that the proposed nomogram, based on routine complete blood counts and initial CT findings, could contribute to individualized prognosis prediction and clinical decision-making in children with MS-TBI.

Coronavirus disease 2019 (COVID-19) affected millions of people worldwide since early 2020 including pregnant women (4). While the majority of neonates born from women with COVID-19 during pregnancy had favorable outcomes, Kobata et al. reported a case of a neonate with grade 2 periventricular leukomalacia (PVL) born from a mother with COVID-19. This report should prompt clinicians to monitor fetal cerebral function and structure immediately after birth.

As for therapeutics in neonatal and pediatric brain injuries, repetitive transcranial magnetic stimulation (rTMS) for treating the motor and language abilities of patients with CP and virtual reality (VR) -based sensory stimulation in treating pediatric disorders of consciousness have been investigated. Sun et al. assessed the effectiveness of rTMS in treating motor and language abilities in CP. Finally, 29 studies were included in the meta-analysis, and the results showed that rTMS improved motor function and language abilities in patients with CP. On the other hand, Liang et al. investigated whether VR-based sensory stimulation could improve the level of consciousness in pediatric disorders of consciousness compared with general rehabilitation. This pilot study indicated the potential benefit of adding VR to standard rehabilitation for pediatric disorders of consciousness. Therefore, large sample randomized controlled trials are needed to obtain sufficient evidence on the effectiveness of new therapeutics for treating pediatric patients.

In conclusion, the papers in this e-book collect contributions from different experts in the field of neonatal and pediatric brain injury and address specific problems, such as novel diagnostic methods, screening tools, and biomolecules for diagnosis, adding new insights and perspectives in the treatment of neonatal and pediatric brain injury.
